# Serological evidence uncovers undocumented mpox exposure in febrile patients in Nigeria

**DOI:** 10.3389/fpubh.2026.1914149

**Published:** 2026-09-15

**Authors:** Jolly A. Adole, Sandra E. Omaga, Precious E. Oyamendan, Utibe M. Dennis, Oluremi I. Ajayi, Amoge C. Ogu, Ifeoluwa O. Bejide, Johnson Okolie, Philomena Eromon, Christian T. Happi, Chinedu A. Ugwu

**Affiliations:** 1 Institute of Genomics and Global Health (IGH), Redeemer’s University, Ede, Nigeria; 2 Department of Biological Sciences, Faculty of Natural Sciences, Redeemer’s University, Ede, Osun, Nigeria

**Keywords:** febrile patients, healthcare setting, Luminex immunoassay, monkeypox virus, Nigeria, public health, seroepidemiology

## Abstract

**Introduction:**

The monkeypox virus (MPXV) has become a global health threat, with rising cases reported globally. Since its re-emergence in 2017, Nigeria has experienced a sustained outbreak, underscoring the critical need for robust surveillance mechanisms to identify transmission dynamics. Despite this urgency, data regarding MPXV seroprevalence among patients seeking medical care in Nigeria remains scarce. MPXV may be underreported because its clinical symptoms overlap with those of other common causes of fever.

**Methods:**

We assess MPXV seroprevalence among febrile patients across multiple Nigerian healthcare facilities using A35R and A29L antigens in a Luminex bead-based immunoassay. The study enrolled 245 febrile individuals in Ondo, Benue, Plateau, and Osun States from June 2023 to July 2024. Plasma samples were analyzed for antibodies targeting MPXV A35R and A29L antigens. A manual-based 3-standard-deviation threshold method and an R-based SeroNIST analytical framework were used. Statistical analyses assessed assay reliability and identified potential risk factors associated with MPXV exposure.

**Results and discussion:**

Both antigens showed comparable exposure patterns (*p* = 0.297). The 3SDV threshold method identified seroprevalence rates of 47.4% for A35R and 40.4% for A29L. The SeroNIST approach yielded estimates of 30.51% and 21.17% for A35R and A29L, respectively. Participants (*n*=62; 25.31%) showed reactivity to both antigens. Bivariate analyses revealed that only the state of residence significantly influenced serostatus for both antigens, whereas sex, occupation, and marital status significantly affected A29L serostatus only. Multivariable regression indicated that none of the variables were significantly associated with A35R seropositivity; however, participants from Ondo showed a higher, but non-significant, odds of seropositivity. For A29L, health workers, males, and participants from Osun had significantly lower odds of seropositivity, while those aged 26–36 had higher non-significant odds. These results confirm considerable MPXV exposure among febrile patients and call for longitudinal and region-specific studies to unravel temporal and geographic trends in its seroprevalence in Nigeria.

## Introduction

Monkeypox virus (MPXV), a zoonotic Orthopoxvirus of the family *Poxviridae*, has re-emerged as a significant public health concern globally, with increasing incidence reported in both endemic and non-endemic regions (1, 2). Historically, MPXV is confined to Central and West Africa (3–5). However, it has gained renewed attention following sustained human-to-human transmission (6, 7), and outbreaks beyond its traditional ecological niche (8). Nigeria has remained one of the countries with ongoing mpox activity since the resurgence of cases reported in 2017 (9, 10) underscoring the need for stronger surveillance systems capable of detecting both symptomatic and asymptomatic infections.

Mpox shares similar febrile clinical symptoms to other common endemic febrile illnesses in Nigeria, including malaria, typhoid fever, and viral hemorrhagic fevers (11, 12). More importantly, it has been reported that a high proportion (147/160; 91.9%) of confirmed mpox cases showed febrile symptoms (11). This may result in clinical misdiagnosis of MPXV infection in healthcare settings. While molecular detection, such as polymerase chain reaction (PCR), remains the gold standard for confirming acute infection, its utility is limited to the narrow window of viral shedding and requires specialized laboratory infrastructure (13, 14). Consequently, many past infections, particularly those with mild or asymptomatic clinical features, remain undetected and could be the sentinel for sustained transmission across the continent. Serological surveillance provides a complementary approach by enabling the detection of past, asymptomatic, or clinically unrecognized ongoing exposure to MPXV, offering valuable insights into population-level exposure and transmission dynamics (15–17).

Public health-guided surveillance has been proven successful in evidence-based decision-making through surveillance data (18). The detection of antibodies in blood is a critical disease surveillance tool since antibodies are typically easier to detect and persist longer than the infectious agents (16). Serological assays detect antibodies induced by infection or vaccination, and provide evidence of past exposure to a pathogen (17). Ecologists, modelers, and policy makers use serological data for inference to pathogen force of infection and transmission rates, as well as to parameterize dynamic disease models (17).

Despite Nigeria’s status as an MPXV-endemic country, limited seroepidemiological data leave several knowledge gaps around the MPXV exposure dynamics. Emerging serological evidence has revealed MPXV exposure in apparently healthy individuals who were previously seronegative, as observed in a longitudinal study with a median follow-up period of 9 months (19). Additionally, several pieces of evidence from advanced settings have uncovered asymptomatic MPXV infections among certain high-risk groups (20–29). Febrile patients represent a unique group, as they can be misdiagnosed with other common causes of fever and may serve as sentinels for disease transmission such as mpox. The dynamics of MPXV among febrile patients presenting to healthcare settings in Nigeria remain poorly understood. Importantly, the extent of MPXV exposure in this population, especially among younger individuals, following the historical discontinuation of smallpox vaccination (30, 31), remains largely unclear.

To address these gaps, we analyzed plasma samples collected from febrile patients across healthcare settings in Nigeria using A35R and A29L antigens of MPXV in a Luminex bead-based immunoassay. Luminex bead-based immunoassay uses relatively small sample volumes to detect antibodies against multiple antigens simultaneously and reduces labor, time, and cost compared with other serological methods such as ELISA (32–34). The A35R and A29L antigens are extracellular enveloped and intracellular mature proteins of MPXV, respectively. These antigens were selected to capture the complementary exposure of the study participants to both extracellular and intracellular immunogenic proteins, providing robust specificity for MPXV or orthopoxviruses (19, 35–39). The seropositivity of both antigens was assessed using the traditional mean + 3SD and the probabilistic SeroNIST framework to provide a more robust and informative strategy. Focusing on the age group 15–36 years, thereby excluding individuals likely to have received smallpox vaccination before its discontinuation in 1980, provides a more accurate assessment of MPXV seropositivity among younger febrile patients without residual smallpox immunity. By leveraging antigen-specific antibody detection, we compared the dynamics of MPXV-specific antibody responses between A35R and A29L in the study population. We further evaluated factors necessary for sustained transmission and exposure. Overall, this study provides evidence of MPXV exposure among febrile patients across healthcare settings in Nigeria, thus contributing to improved surveillance frameworks and supporting the integration of serology into routine febrile illness investigations in MPXV-endemic settings.

## Materials and methods

### Ethical clearance

All methods were carried out in accordance with relevant guidelines and regulations. All subjects enrolled in this study and/or their guardians provided informed consent. Human subjects testing and sample collection were approved by the Redeemer’s University Institutional Review Board, and the Nigerian National Health Research Ethics Committee (NHREC/01/01/2007–11/09/2023).

### Study hospitals

This multicentre cross-sectional seroepidemiological study was conducted from June 2023 to July 2024 at 15 study sites in 4 States of Nigeria including (i) Plateau State (Vom Christian Hospital (VCH)), (ii) Benue State (The Federal University of Health Science Teaching Hospital Otukpo, Primary Health Care I Otukpo, Primary Health Care II Otukpo, Saint Veronica Clinic & Maternity Otukpo, Royal Specialist Hospital Otukpo and Saint Daniel Clinic Otukpo), (iii) Ondo State (Federal Medical Center Owo, Sckye Hospital Akure, First Mercy Hospital Akure, My Shepherd Hospital Akure, Foundation Specialist Hospital Akure, Federal Medical Center (Annex) Akure and Mao Hospital Akure), and (iv) The Redeemer’s University Health Center (RUNHC), Ede, Osun State. These study sites are located in North Central and Southwestern Nigeria (Figure 1). The choice of these sites was based on convenience and the hospital authorities’ willingness to collaborate with the study team. Additionally, cultural practices such as farming and hunting activities, including previously reported outbreaks in the participants’ states of residence, were among the factors that informed the selection of the study sites (40, 41). Field teams were trained in Good Clinical Practice (GCP), biosafety procedures, and standardized data collection methods. Community engagement was conducted at each site to ensure culturally appropriate, voluntary participation. The study design ensured contextual relevance and operational feasibility.

**Figure 1:**
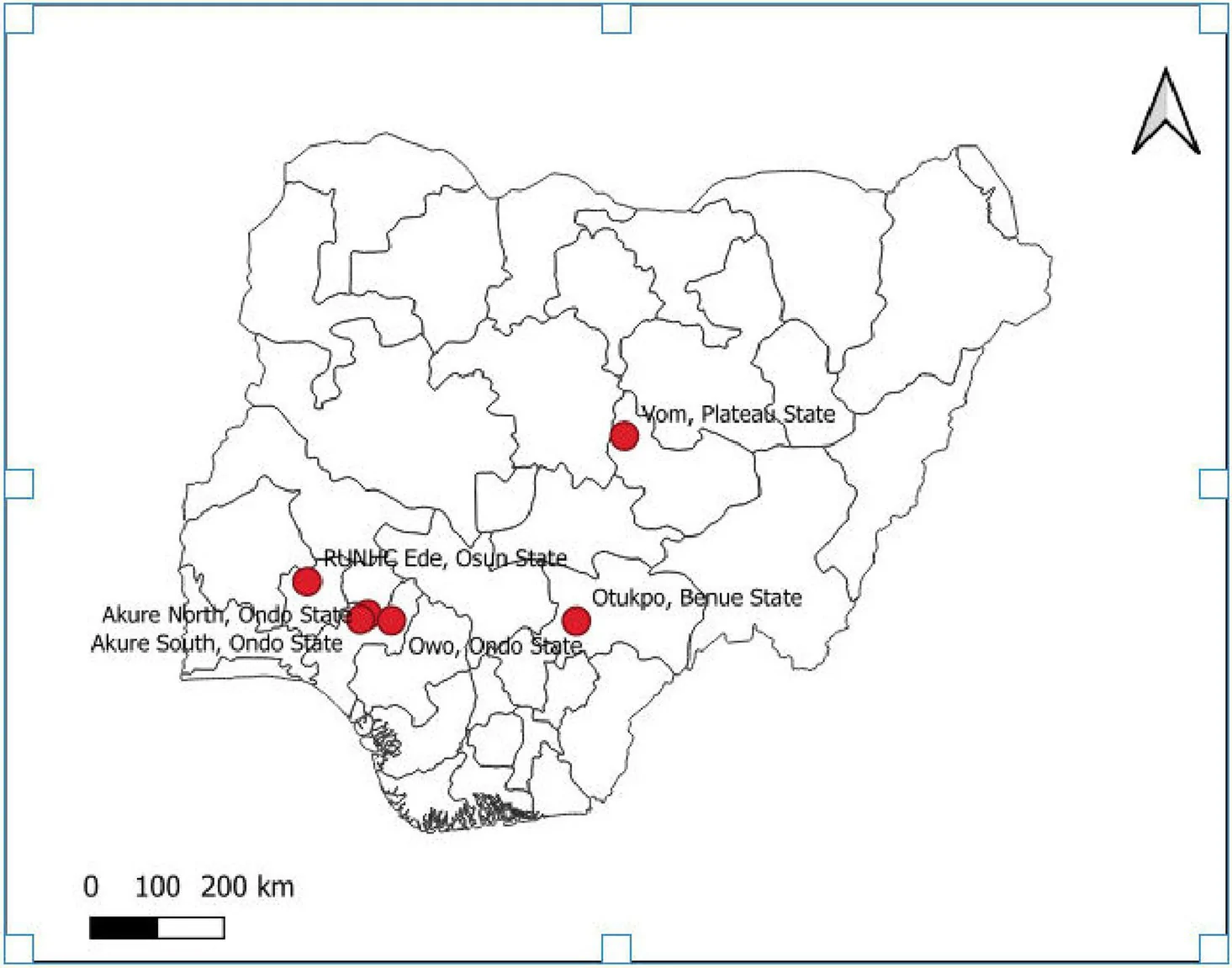
Map of Nigeria showing the study areas (red dots).

We used convenience sampling to recruit febrile patients from several hospitals based on feasibility, logistical constraints, available funding, and the anticipated number of eligible febrile patients attending participating hospitals during the study period. A total of 245 participants were recruited for the study, from RUN Health Center Ede, Osun State (*n* = 32), Akure North (*n* = 40), Akure South (*n* = 28), and Owo (*n* = 40), in Ondo State from April to June 2024, and from Otukpo Benue State (*n* = 74) from May to June 2024 and Vom, Plateau State (*n* = 31) in July 2024.

### Inclusion criteria

This study included febrile patients aged 15–36 years, of all genders, who agreed to participate after a clear understanding of the project and signing of the informed consent form. This age range was particularly chosen for this study to exclude all individuals who were likely to have been vaccinated against the smallpox virus in 1980, when smallpox vaccination was discontinued (30, 31). However, participants under 18 years old were enrolled if their parents/guardians signed the informed consent on their behalf. The study’s purpose, objectives, and potential benefits were explained to the participants. Once they provided consent by signing the informed consent form, febrile patients who met the inclusion criteria completed the survey and provided 5-10 mL of blood samples.

### Exclusion criteria

Individuals with the following health conditions, such as those in the intensive care unit, pregnant women, severe respiratory cases, prisoners, those with psychosis, severe cancer patients, and patients in a coma, were not recruited. Also, people over 36 years and those unwilling or unable to provide informed consent were excluded.

### Plasma separation

The plasma was separated as follows: 5–10 mL of whole blood was transferred from the EDTA tubes into a LeucoSep tube containing Ficoll–Hypaque at a ratio of 2:1. The tube was centrifuged at 800 x g for 30 min at room temperature in a swinging-bucket rotor with no brake, and the top layer of plasma was removed (42). The plasma was transferred to 2 mL cryotubes and stored at −20 °C until needed for further analysis.

### MPXV antigens used in this study

The recombinant Monkeypox virus antigenic proteins used for this study include A35R (Monkeypox Virus strain Zaire-96-I-16, PVA001) and A29L (Monkeypox Virus MPXV_USA_2022_MA001, PVA005), UniProt Accession No.: Q8V4U4 and Q90188, respectively. The A35R protein was constructed using the sequence of an MPXV isolate obtained from a patient during the large human monkeypox outbreak in Zaire (Democratic Republic of Congo) in 1996 (43), whereas the A29L protein was constructed from the MPXV isolate responsible for the 2022 multi-country outbreak in the United States (44). These proteins were kindly provided by https://www.sanyoubio.com (Sanyou Biopharmaceuticals Co., Ltd., China). Our antigen selection was guided by previous work, which identified A29L and A35R core immunodominant antigens in response to infection and vaccination by mpox and vaccinia viruses (39, 45–48).

### Bead coupling and Luminex bead-based serology

The antigen-specific antibody responses were quantified using a previously validated Luminex bead-based immunoassay protocol, which enables the simultaneous detection of antibodies in a single sample (34). Briefly, the target antigens include MPXV A35R and A29L, and Bovine serum albumin (BSA) proteins. The proteins were reconstituted appropriately following the manufacturer’s instructions. The MPXV A35R and A29L protein antigens were coupled separately to the MagPlex microspheres (Luminex Corporation). BSA-coupled beads were used to control for bead-specific background noise. All coupled beads were diluted in ABE buffer (PBS, BSA 0.1%, TWEEN 20%, Sodium Azide 0.05%) to make a master mix of each coupled antigen and the coupled BSA (consistent concentration across all beads; 1,000,000 beads/mL was maintained). Then, 50 μL of human plasma samples diluted (1:100) in ABE buffer were added in duplicate to the wells of a 96-well plate. Briefly, we performed a fourfold serial dilution of the positive controls (standard) and included blank wells in the assay plate layout for quality check. Subsequently, 50 μL of bead master mix was added to each well of the 96-well plate and followed by a 2-h incubation period on a plate shaker (300 rpm). Next, the beads were washed three times with 125 μL ABE buffer while the plate remained on the magnet. Then, 50 μL of biotinylated anti-human secondary IgG (BD Pharmingen) diluted in ABE (1:1000) was added to each well and followed by a one-hour incubation at room temperature on a plate shaker (300 rpm). The beads were once again washed three times, and 50 μL of phycoerythrin-conjugated streptavidin (1:1000; BD Pharmingen catalog # 554061) diluted in ABE was added to the wells. Next, the beads were set on the plate shaker at 300 rpm for 15 min. After the final incubation period, the beads were washed 3 times for the last time and resuspended in 75 μL of ABE. After resuspension, the plate was read on the Luminex MagPix instrument, where a median fluorescence intensity (MFI) value was generated for each sample and individual antigen with the corresponding bead count. Then, the control MFIs (positive and negative controls), standard (dilution of positive control), and bead counts (minimum 50 beads per antigen in each well) were validated. We then applied normalization across plates based on the positive control and BSA MFI signals, generated a cutoff MFI, and transformed the MFIs into qualitative positive and negative outcomes (49, 50). To normalize the data, the background noise was removed using the BSA MFI values for each sample. A schematic overview of the Luminex xMap-based serological assay workflow is shown in Figure 2. Previously collected de-identified plasma samples banked at the Institute of Genomics and Global Health, Redeemer’s University, Ede, Nigeria, served as negative and positive controls. We used positive control samples (*n* = 4) to generate multiple MFI signals used in the R-based SeroNIST-style framework method, whereas the negative control plasma samples (*n* = 18) were obtained from healthy individuals.

**Figure 2:**
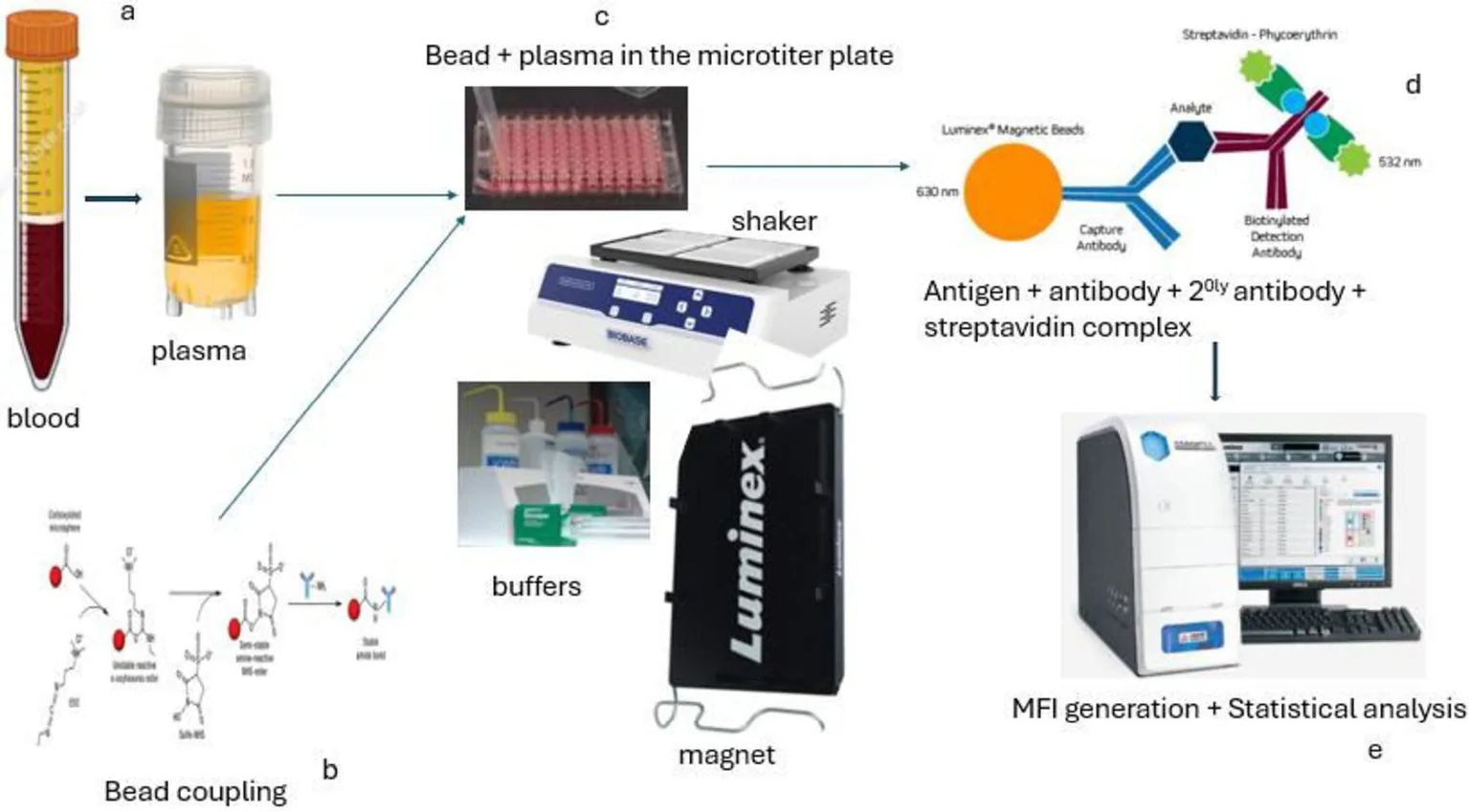
Schematic overview of the Luminex xMAP-based serological assay workflow used: **(a)** Isolation of plasma from the whole blood by centrifugation, **(b)** Preparation of MagPlex microsphere (beads) including washing, activation, and covalent coupling of the beads with the recombinant MPXV A35R and A29L antigens, **(c)** Assay setup, in which 50 μL of diluted plasma (1:100) and 50 μL of the antigen-coupled beads master mix were added in duplicate to each well of a 96-well microtiter plate, followed by sequential incubation, shaking, magnetic washing, and addition of a biotinylated secondary antibody and streptavidin-phycoerythrin (SAPE) reporter. **(d)** Principle of antibody detection: In seropositive samples, MPXV-specific antibodies bind to the antigen-coated beads, forming immune complexes that are recognized by the secondary antibody and subsequently detected through SAPE-generated fluorescence. **(e)** Acquisition and quantification of fluorescence as mean fluorescence intensity (MFI) using the Luminex xMAP platform for statistical analysis.

### Threshold assessment methods 3STDV and R-based SeroNIST framework

We determined the A35R and A29L antigens seropositivity cut-off values among study participants using two different methods to provide a more robust and informative strategy. We compared two different methods to calculate thresholds and determine which samples are considered positive or negative according to the calculated cut-offs: (1) The first approach was a traditionally applied method where the threshold is set at three standard deviations above the mean of the negative controls (3STDV). (2) The second approach was based on a standardized R-based SeroNIST-style framework method that used Log-transformed MFI values from negative and positive controls to model antibody signal distributions. For each sample, the probability of seropositivity was calculated, and population-level seroprevalence was estimated as the mean of individual probabilities, with uncertainty assessed via bootstrap resampling. It calculates the probability of seropositivity, seroprevalence, and the 95% uncertainty interval (Table 1). It used four positive control samples (MFI signals, *n* = 18) generated from multiple runs, 18 negative control signals (*n* = 18), and the study samples’ signals (*n* = 245) to model the signal distribution (fitting a normal distribution to controls) after log transformation of the raw signals (Figures 3c,d).

**Table 1:** Mpox seroprevalence rate among participants by the threshold methods.

MPXV Antigens	Total samples	3STDV	SeroNIST	
		Seroprevalence %	Seroprevalence %	95% uncertainty interval (2.5–97.5%)
A35R	245	47.4	30.51	25.16–35.77
A29L	245	40.4	21.17	16.75–25.65

**Figure 3:**
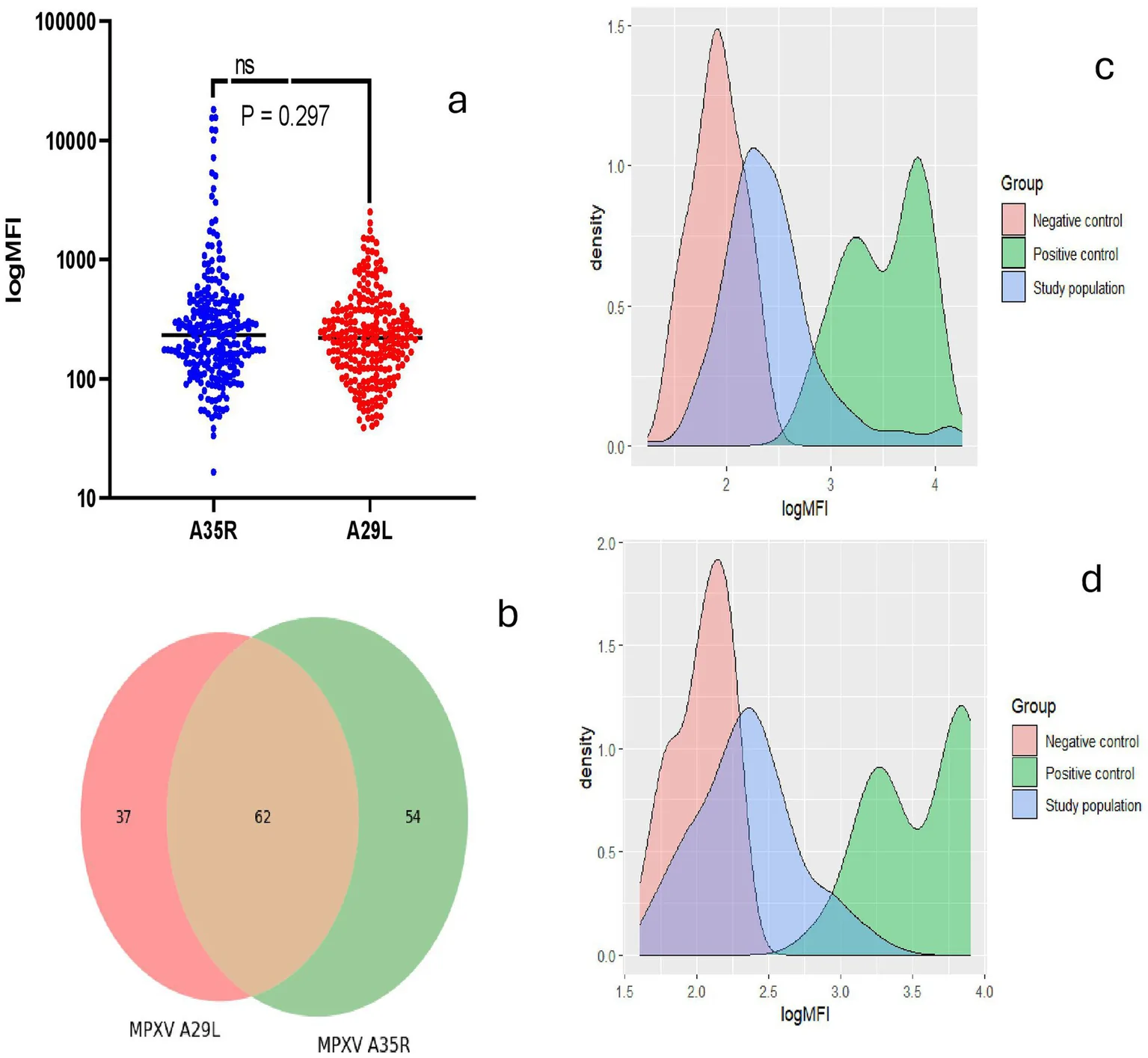
**(a)** Comparable antibody responses to MPXV A35R and A29L antigens. **(b)** Venn diagram showing overlap and unique antibody reactivity to MPXV A35R and MPXV A29L antigens, **(c)** distinct log MFI signal distributions of A35R antigens between negative, positive, and study groups, and **(d)** distinct log MFI signal distributions of A29L antigens between negative, positive, and study groups.

### Statistical analysis

Statistical calculations and visualizations were performed using Prism (version 8.2.1), Python version 3.13.5 packaged by Anaconda, Inc., and R (version 4.5.1). We applied the threshold formula: mean + 3 standard deviations of negative controls (50) and an R-based SeroNIST-style workflow for cutoff determination and assay validation. Log-transformed MFI values from negative and positive controls were used to model antibody signal distributions. For each sample, the probability of seropositivity was calculated and population-level seroprevalence estimated as the mean of individual probabilities, with uncertainty assessed via bootstrap resampling with a fixed random seed. Briefly, due to limited resources, we used positive control signals (*n* = 18) generated from repeated runs of four known positive samples, whereas the negative control signals (*n* = 18) were plasma samples obtained from healthy individuals, and the study samples’ signals (*n* = 245) to model the signal distribution (fitting a normal distribution to controls) after log transformation of the raw signals (Figures 3c,d). Then the probability of seropositivity, seroprevalence, and the 95% uncertainty interval were computed for each antigen (Table 1). All the controls were previously collected de-identified plasma samples banked at the Institute of Genomics and Global Health, Redeemer’s University, Ede, Nigeria.

Pearson’s Chi-squared test with Yates’ continuity correction and Fisher’s exact test were used to assess the association between the sociodemographic variables and the seropositivity of the study participants. The variables, including age, sex, occupation, geolocation, state of residence, and marital status, were selected for analysis based on biological plausibility and epidemiological relevance. Our assessment for multicollinearity and overall model fit indicated that the multivariable logistic regression model was well specified, demonstrated adequate fit, and showed no evidence of problematic multicollinearity. Additionally, the ROC curve indicated that the model discriminated between A35R (AUC = 0.69) and A29L (AUC = 0.70) seropositive and seronegative individuals better than chance (Supplementary Figures 2, 3; Supplementary files). Hence, logistic regression was performed on MPXV A35R and A29L serology (positive or negative for anti-MPXV antibodies) in RStudio using the glm function. We created a visualization plot of anti-MPXV A35R and A29L antibody binding signal among study participants, using the raw median fluorescence intensity (MFI). The Differences between anti-MPXV A35R and A29L antibody MFI values were assessed with the unpaired nonparametric Mann–Whitney U test. The graph (Figure 3a) was on a Log 10 scale to visualize the MFI spread, and the statistical test was applied to raw/non-transformed numbers. Also, we performed Cohen’s Kappa agreement and Spearman’s ranked Correlation analysis between A35R and A29L antigens (Supplementary Figure 1).

## Results

### Study population and demographics

Over half of the participants were female (*n* = 156, 63.7%), while 36.3% (*n* = 89) were male. The study comprised participants aged 15 to 36. The majority of participants were students (*n* = 90), followed by self-employed individuals (*n* = 76), healthcare workers (*n* = 46), civil servants (*n* = 19), and farmers (*n* = 14). Married and single participants were *n* = 85 and *n* = 160, respectively. The highest number of participants was from Ondo State (*n* = 108), followed by Benue State (*n* = 74), Osun State (*n* = 32), and Plateau State (*n* = 31). On the other hand, the number of study participants from rural and urban settings is 73 and 172, respectively (Tables 2, 3).

**Table 2:** Bivariate analysis of risk factors associated with MPXV A35R antigen seropositivity.

Variable	Category	n(%)	n positive (%)	n negative (%)	χ^2^ (df)	*p*-value
Sex	Male	89(36.33)	39(43.82)	50(56.18)	0.49(1)	0.483
	Female	156(63.67)	77(49.36)	79(50.64)		
Age	15–25	147(60.00)	64(43.54)	83(56.46)	1.77(1)	0.183
	26–36	98(40.00)	52(53.06)	46(46.94)		
Marital status	Single	160(65.31)	66(41.25)	94(58.75)	6.190(1)	0.0129
	Married	85(34.69)	50(58.82)	35(41.18)		
Geolocation	Rural	73(29.80)	42(57.53)	31(42.47)	3.77(1)	0.052
	Urban	172(70.20)	74(43.02)	98(56.98)		
State of residence	Plateau	31(12.65)	16(51.61)	15(48.39)	Fisher’s exact	2.353e-09
	Benue	74(30.20)	44(59.46)	30(40.54)		
	Ondo	108(40.08)	56(51.85)	52(48.15)		
	Osun	32(13.06)	0(00.00)	32(100.00)		
Occupation	Farming	14(5.71)	7(50.00)	7(50.00)	9.36(4)	0.053
	Student	90(36.73)	33(36.67)	57(60.33)		
	Healthcare worker	46(18.78)	23(50.00)	23(50.00)		
	Self Employed	76(31.02)	44(57.89)	32(42.11)		
	Civil Servant	19(7.76)	9(47.37)	10(52.63)		

X^2^ = Chi-square statistic and degree of freedom; n(%) = total count and percentage.

**Table 3:** Bivariate analysis of risk factors associated with MPXV A29L antigen seropositivity.

Variable	Category	n(%)	n positive (%)	n negative (%)	χ^2^ (df)	*p*-value
Sex	Male	89(36.33)	27(30.34)	62(69.66)	5.25(1)	0.023
	Female	156(63.67)	72(46.15)	84(53.85)		
Age	15–25	147(60.00)	54(36.73)	93(63.27)	1.70(1)	0.193
	26–36	98(40.00)	45(45.92)	53(54.08)		
Marital status	Single	160(65.31)	56(35.00)	104(65.00)	4.97(1)	0.026
	Married	85(34.69)	43(50.59)	42(49.41)		
Geolocation	Rural	73(29.80)	28(38.36)	45(61.64)	0.081(1)	0.776
	Urban	172(70.20)	71(41.28)	101(58.72)		
State	Plateau	31(12.65)	11(35.48)	20(64.52)	14.76(3)	0.002
	Benue	74(30.20)	43(58.11)	31(41.89)		
	Ondo	108(40.08)	37(34.26)	71(65.74)		
	Osun	32(13.06)	8(25.00)	24(75.00)		
Occupation	Farming	14(5.71)	6(42.86)	8(57.14)	12.49(4)	0.0141
	Student	90(36.73)	34(37.78)	56(62.22)		
	Health worker	46(18.78)	10(21.74)	36(78.26)		
	Self Employed	76(31.02)	38(50.00)	38(50.00)		
	Civil Servant	19(7.76)	11(57.89)	8(42.11)		

X^2^ = Chi-square statistic and degree of freedom; n(%) = total count and percentage.

### MPXV seroprevalence among participants

Seropositivity was estimated by two methods: the manual three-standard-deviation (mean + 3SDV) method and the R-based SeroNIST-style framework. For the manual method, cutoff values were defined based on the distribution of negative control samples. For each antigen, the cutoff was calculated as the mean of the median fluorescence intensity MFI of negative controls plus three standard deviations (mean + 3SDV), a conservative threshold chosen to minimize false-positive classification. The calculation of the threshold using the mean plus three standard deviations (mean + 3SD) yielded a value of 244.11, based on a mean of 92.49 and a standard deviation of 50.54 for A35R, and a value of 254.66 based on a mean of 116.0 and a standard deviation of 46.22 for A29L. Using the mean + 3 SDV approach, the seropositivity cutoff was calculated at MFI cutoffs of ≥ 244.11 and ≥ 254.66 for A35R and A29L, respectively. Study samples with MFI values ≥ 244.11 and 254.66 were classified as seropositive, yielding seroprevalence estimates of 47.4 and 40.4% for A35R and A29L, respectively.

For the R-based SeroNIST-style framework approach, the posterior probabilities of seropositivity were calculated for study samples. The estimated seroprevalence of A35R and A29L-specific antibodies binding in the study population was 30.51 and 21.17%, with 95% uncertainty intervals derived from bootstrap resampling equal to 25.16–35.77% and 16.75–25.65%, respectively (Table 1). A total of 54 samples reacted only to MPXV A35R, while 37 samples reacted only to MPXV A29L. In addition, 62 (25.31%) samples showed reactivity to both antigens, indicating a shared response to MPXV A35R and A29L (Figure 2b). Cohen’s Kappa result, κ = 0.25, falls within the fair agreement range (0.21–0.40), and a *p*-value of 0.00008 and Spearman’s rho (ρ) = 0.329; *P*-value = 1.34 × 10^−7^ (0.000000134) indicate a weak to moderate relationship between A35R and A29L antigens.

### Analysis of possible determinants of MPXV seropositivity

Using bivariate analysis, we assess the relationship between each variable (sex, occupation, geolocation, marital status, and state of residence) and seropositivity to MPXV antigens using Pearson’s Chi-Square with Yates’ continuity correction and Fisher’s Exact test. There was a statistically significant relationship between A29L seropositivity and sex, occupation, state of residency, and marital status (married vs. single). Similarly, a statistically significant correlation was found between seropositivity to MPXV A35R antigens and the state of residency, including marital status. On the other hand, there was no significant correlation between A35R seropositivity and sex, age, occupation, and geographic area (rural vs. urban). Similarly, age, including geographic area (rural vs. urban), did not exhibit a significant association with seropositivity to A29L MPXV antigen (See Tables 2, 3).

We conducted a multivariable logistic regression to explore whether factors like sex, age, occupation, geographical location, state of residence, and marital status were independently linked to the odds of being seropositive for the MPXV A35R and A29L antigens. For A29L, we found that males, healthcare workers, and participants from Osun State had significantly lower odds of seropositivity compared to their reference group. Similarly, farmers and self-employed individuals, along with participants from Ondo and Plateau States, showed non-significantly lower odds of A29L seropositivity. However, individuals aged 26–36 years and those living in urban areas had non-significantly higher odds of A29L seropositivity compared to their younger counterparts aged 15–25 years and those in rural areas, respectively. On the other hand, none of the variables showed a significant association with the odds of being seropositive for the A35R antigen. However, there were higher, non-significant odds of being seropositive for participants from Ondo State, while those from Osun State appeared to have lower, but non-significant odds (see Figures 4, 5) and the Supplementary file.

**Figure 4:**
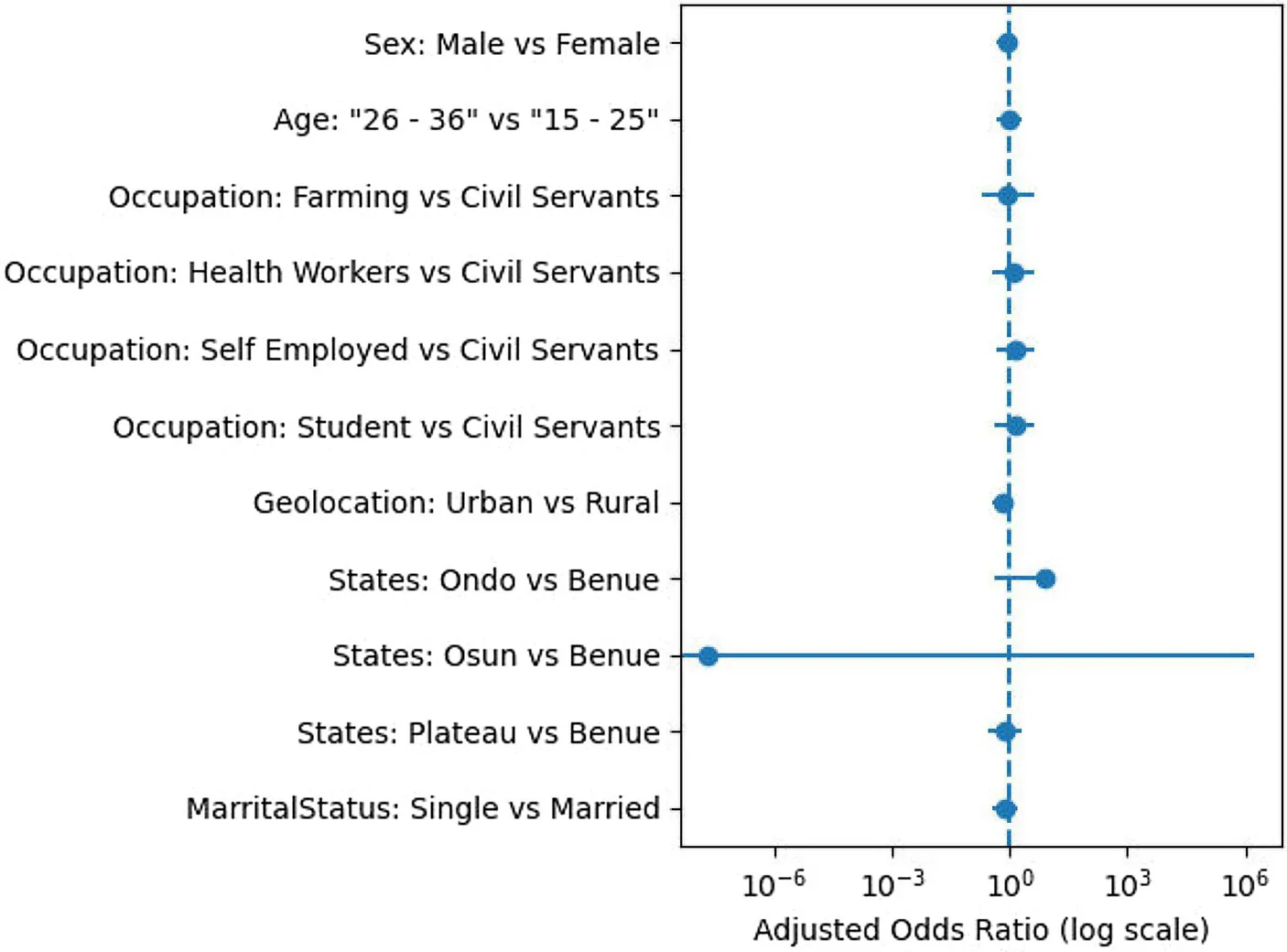
Forest plot of multivariable logistic regression analysis results of MPXV A35R antigen. The blue dot is the adjusted odds ratio (AOR), the horizontal line is the 95% confidence interval (CI), and the vertical dashed line at AOR = 1 represents “no association” (AOR > 1: igher odds of the outcome compared with the reference group and AOR < 1: lower odds of the outcome compared with the reference group). If the CI crosses 1, the association is not statistically significant at the 5% level.

**Figure 5:**
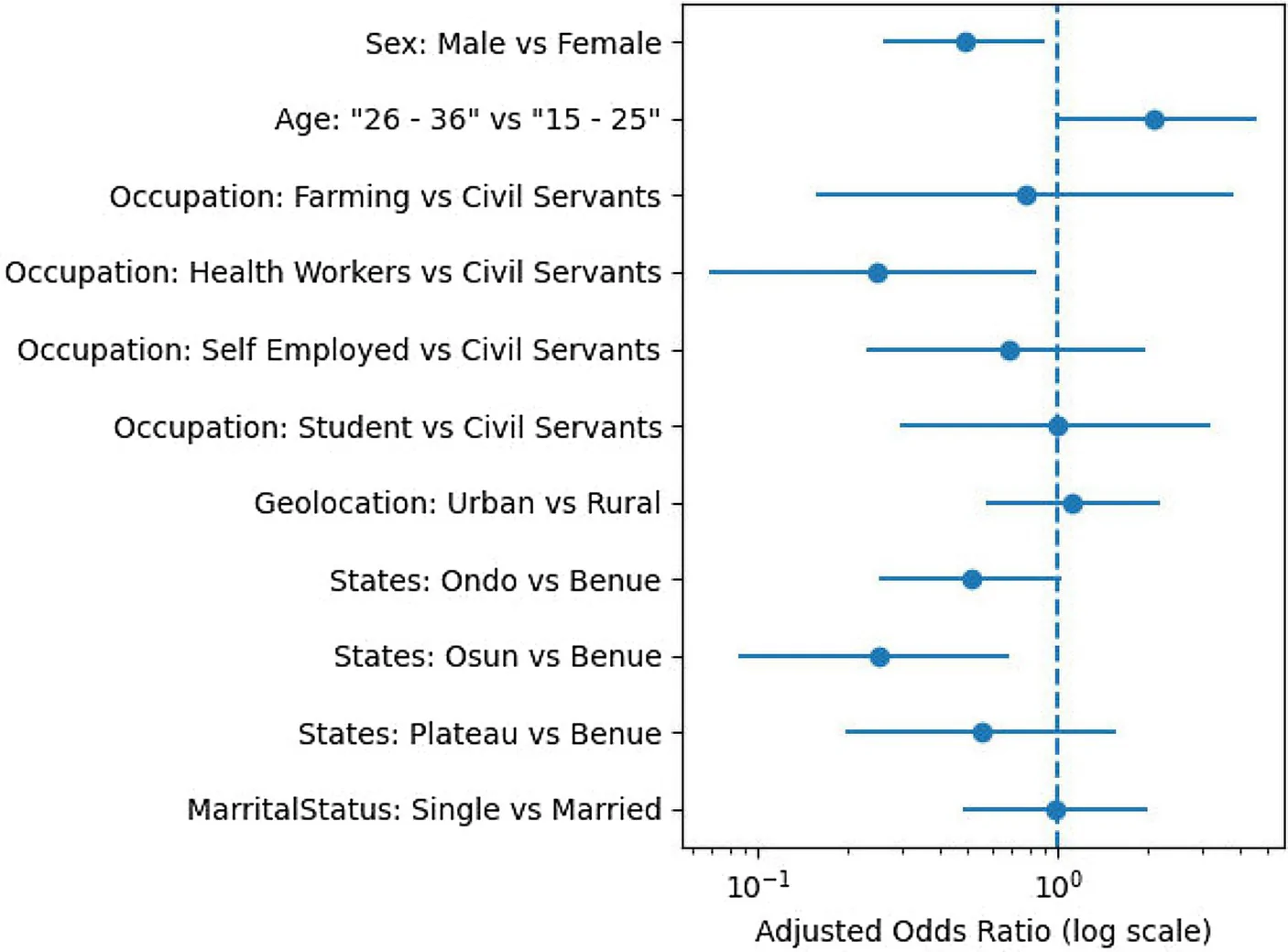
Forest plot of multivariable logistic regression analysis results of MPXV A29L antigen. The blue dot is the adjusted odds ratio (AOR), the horizontal line is the 95% confidence interval (CI), and the vertical dashed line at AOR = 1 represents “no association” (AOR > 1: Higher odds of the outcome compared with the reference group and AOR < 1: Lower odds of the outcome compared with the reference group). If the CI crosses 1, the association is not statistically significant at the 5% level.

## Discussion

MPXV has re-emerged as a significant public health concern globally, with increasing incidence reported in both endemic and non-endemic regions (1, 2). Nigeria has remained one of the countries with ongoing mpox activity since the resurgence of cases (9, 10), underscoring the need for strengthened surveillance systems capable of detecting evidence of exposure to infection. Serological surveys provide the most direct measurement of exposure to many infectious diseases (16). Population-level exposure can be assessed by detecting antibodies in blood samples from the target population. Serological tests detect an antibody response to infection or vaccination; they indicate past or current exposure to a pathogen (17). Moreover, pathogen-specific antibodies usually last longer than the causative pathogen and are easier to find (16). Although Nigeria is an MPXV-endemic country, there is limited data on MPXV seroprevalence among people seeking healthcare. Febrile patients are especially important to monitor. They often visit healthcare facilities and can signal an ongoing infection. This study provides evidence of Monkeypox virus (MPXV) seroprevalence among febrile patients from multiple healthcare facilities in Nigeria, as measured using A35R and A29L antigens in a Luminex serological assay. Sampling across four states improves the validity of our findings compared with other single-site studies in Nigeria. The findings provide insight into MPXV exposure among healthcare-seeking populations, underscoring the continued circulation of MPXV in Nigeria beyond clinically confirmed cases.

### Study population and demographics

Previous MPXV serological studies focused on the general population across all ages, including young and old participants (19, 51). However, our study is unique because it focused on younger participants (15 to 36 years) who were born after the discontinuation of smallpox vaccination in 1980 (30, 52), thereby reducing the likelihood of cross-reactivity with residual immunity from smallpox vaccination in older people. Most of our study population were between 15 and 25 years of age, and we found that female participation was higher, similar to Olayiwola et al. (51), and differs from Ogoina et al. and Quiner et al. (11, 53). Our finding suggests that women and socially active populations engage more in public health research, and this aligns with previous studies (34, 54, 55). The overrepresentation of females in our study implies that the interpretation may be limited in generalization to the broader population. Furthermore, across occupation categories, the majority of participants were students, while Ondo State had the highest number of participants in the state-of-residence category, and single individuals outnumbered married individuals in our study. This variation in the demographic participation trend is consistent with other previous studies (11, 51, 53). Differences in the social, caregiving, ethnic, and religious factors, including levels of trust, willingness, and motivation to participate, may have contributed to the overrepresentation of certain demographic groups in our study, which aligned with the previous reports identifying these factors influencing participation in public health studies (46, 56–59).

### MPXV seroprevalence among febrile patients in Nigeria

In this study, we found MPXV seroprevalence of 47.4 and 40.4% for A35R and A29L, respectively, using the “mean + 3SDV” threshold method. To ensure robustness, we performed a probabilistic estimate of seroprevalence for A35R and A29L using an R-based SeroNIST-style framework, yielding estimates of 30.51 and 21.17%, with bootstrapped 95% uncertainty intervals of 25.16 to 35.77% and 16.75 to 25.65%, respectively. The uncertainty interval reflects both biological variability in antibody responses and sampling uncertainty, indicating that the true seroprevalence lies within this interval. The limited number of positive and negative controls may reduce the statistical power, and the estimated distributions may not fully represent the variability in antibody responses across the broader population. Nevertheless, the observed seroprevalence in this study is consistent with previous reports indicating ongoing MPXV transmission in Nigeria, including both recognized outbreaks (41, 56) and unrecognized community exposure (19). Most previous studies focused on general populations, outbreak cohorts, and certain risk groups (11, 19, 27, 29, 51, 57, 58); however, studying febrile clinic patients as in our case is novel and useful for public health intervention strategies. Since the re-emergence of mpox in Nigeria in 2017, multiple studies have documented sustained transmission, often with underreporting of the true burden (9, 10, 41, 56, 59); possibly due to reliance on clinical case definitions and limited laboratory confirmation.

The detection of antibodies to both A35R and A29L antigens, associated with intracellular mature and extracellular enveloped virion particles, supports MPXV exposure rather than transient or nonspecific antibody responses (37–39). Given that Cohen’s Kappa, κ = 0.25, which falls within the fair agreement range (0.21–0.40), and the *p*-value of 0.00008 indicates that the observed agreement is significantly greater than would be expected by chance alone. In other words, there is statistical evidence that the two antigens are associated, although the magnitude of agreement remains relatively weak. Similarly, there is a statistically significant positive correlation between the two antigens: Spearman’s rho (ρ) = 0.329; *P*-value = 1.34 × 10^−7^ (0.000000134). This implies that, as one antigen increases, the other tends to increase as well. However, the strength of the association is weak to moderate, indicating that the relationship is present (See supplementary Figure 1).

Additionally, the observed variation between A35R and A29L seroprevalence in our study aligned with other previous studies that combined multiple MPXV antigens and demonstrated different degrees of antibody binding affinity among MPXV antigens (19, 29, 39, 60). Although A35R and A29L are immunodominant MPXV antigens, absolute discrimination from other orthopoxvirus exposure cannot be guaranteed in serological assays. We show both distinct and overlapping seropositivity to MPXV A35R and MPXV A29L antigens (Figure 3b), highlighting their value as sensitive markers for MPXV exposure or serological surveillance, and potential therapeutic targets (39).

### Risk factors for MPXV seropositivity

Previous studies have highlighted factors that could be driving MPXV exposure and disease outbreak, such as behavioral or demographic factors, reservoir hosts, and ecological factors in Nigeria and globally (19, 56, 61, 62). According to our data, seropositivity to both antigens A35R (*p* = 2.353e-09) and A29L (*p* = 0.002) was strongly associated with the state of residency. This finding corroborates previous reports that identified specific geographical locations as risk factors for MPXV transmission in Nigeria (40, 51, 56, 63). The observed variation may reflect differences in distribution of reservoir hosts; forested ecology, human-animal interaction, and behavioral factors that enhance MPXV exposure dynamics. As a limitation, we acknowledged that our study did not include participants’ exposure history, such as contact with infected individuals, animals, and travel history; consequently, region-specific surveillance may be needed to elucidate these factors.

Similarly, occupation is significantly associated with A29L seropositivity, suggesting that certain occupations may be a risk factor in this setting, though contrary to the previous finding by Abdullahi et al. (19). However, our finding implies that there is work-related risk for MPXV exposure, which aligns with another previous study by Szkiela et al. (64). For instance, a case of occupational exposure to mpox has been reported in a 31-year-old previously healthy male with mpox infection following a needle stick injury with a sterile needle through a contaminated glove (65).

The difference between the two MPXV antigens in the statistical analysis showed that, together, the results highlight the importance of using multiple antigens in sero-epidemiological studies. This finding corroborates a previous study (66) since antigen-specific differences in antibody kinetics, durability, and cross-reactivity can influence the detection of exposure patterns (67–70). Hence, the inclusion of A35R and A29L antigens in our study improved exposure detection by capturing a broader spectrum of antibodies, as in the earlier studies (19, 34) See (Tables 1–3).

Furthermore, multivariable logistic regression analysis indicates that males, health workers, and participants from Osun had significantly lower odds of seropositivity to the A29L antigen. Also, participants aged 26–36 years were more likely to be seropositive to the A29L antigen, but not statistically significant. In contrast, none of the variables were significantly associated with seropositivity to MPXV A35R antigens. This finding is in contrast to previous reports documenting predominant MPXV in males, particularly among men who have sex with men (58, 71, 72, 75, 76). The conservatively estimated low rate of less than 1% of men who have sex with men in Nigeria (73, 74, 77, 78), as well as fewer men compared to female participants in our study, may contribute to the low odds of the male gender. Hence, the low observed odds among males should be interpreted cautiously, as the finding may reflect relative underrepresentation of male participants, and the conservatively estimated low proportion of men who have sex men. Consequently, the statistical power to explain this association may have been reduced. This could also suggest that the exposure patterns among febrile healthcare attendees may differ from those observed in outbreak-focused surveillance.

The low odds in healthcare workers may reflect stronger infection prevention practices, heightened awareness, and the potential effectiveness of infection control measures in reducing occupational exposure (79, 80). Our study findings may indicate that the heightened fear of occupational exposure to MPXV in healthcare facilities reported in previous studies (68, 69, 81) are likely to be context-dependent.

The age group 26–36 with high odds of A29L seropositivity suggests their higher involvement in mobility, occupational activity, and social interaction, increasing their risk for MPXV exposure (82, 83). Importantly, the age group 26–36 in our study comprises individuals who are unlikely to have received childhood smallpox vaccination, suggesting that detected antibodies are more likely due to natural exposure rather than smallpox vaccine-derived immunity (19).

The higher odds of A35R seropositivity in Ondo may be consistent with MPXV cases and exposure in the region (40, 41). In contrast, the reduced A29L seropositivity in Ondo, Osun, Benue, and Plateau suggests that not all markers of exposure behave similarly across-the-board (19, 34, 47). Ondo State has a substantial rural population engaged in activities such as farming and hunting, hence faces a heightened risk of exposure to rodents and other animal reservoirs of MPXV (84, 85). Ecological differences, population density, animal reservoirs, and prior outbreak history may also explain the geographic differences in the patterns of seropositivity observed in our study states (61, 86–88) (See Supplementary Tables 2, 3).

### The serological threshold assessment comparison

Our study compared two threshold calculation methods to evaluate the seroprevalence. There was a slight difference between the results of the two approaches. The R-based SeroNIST-style framework method uses Log-transformed MFI values from negative and positive controls to model antibody signal distributions. This method calculates thresholds by utilizing measurements from positive and negative controls. We used a limited number of positive and negative controls, with positive control signal values derived from repeated measurements of only four samples, which increases the accuracy of the assay. However, the estimated distributions may not fully represent the variability in antibody responses across the broader population. Furthermore, fitting a normal distribution to log-transformed signals assumes that log-transformed data were normally distributed; however, this assumption may not be entirely met by the actual data, potentially affecting seropositivity classification. On the other hand, the 3STDV approach is based on only the negative controls. Overall, the two methods estimated similar seroprevalence outcomes. However, the SeroNIST method represents a more rigorous and standardized approach. It automatically calculates seroprevalence and the associated uncertainty estimates (95% Uncertainty Interval) for the population-based and individual serological outcomes. Using both standardized approaches will make the seroprevalence calculations more intensive, informative, and comparable across our study, making the outcomes more suitable for meta-analysis and increasing the public health impact of serosurveys overall (34). Hence, we considered the two approaches- 3STDV and R-based SeroNIST method as complementary for a more robust and informative strategy. The calculation of the threshold using the mean plus three standard deviations (mean + 3SD) yielded a value of 244.11, based on a mean of 92.49 and a standard deviation of 50.54 for A35R, and a value of 254.66 based on a mean of 116.0 and a standard deviation of 46.22 for A29L. This approach is commonly used in immunoassay analyses to define a cut-off background signal and to distinguish true positive responses from negative or nonspecific reactivity (50). While the mean + 3SD method is conservative and minimizes the likelihood of false-positive classification, the wide dispersion reflected by the standard deviation yields a relatively high cut-off value. This may reduce assay sensitivity by potentially classifying low-level true antibody responses as negative (50). To improve the robustness and reliability of cut-off estimation (34), we adopted an R-based SeroNIST-style framework to estimate seropositivity and seroprevalence. The signal distribution plot clearly differentiates between the negative and positive control groups. At the same time, the study population exhibits an intermediate distribution, consistent with varying levels of exposure within the population, supporting the suitability of A35R and A29L antigens for serological assays (Figures 3c,d).

### Strengths, limitations, and implications

Several earlier studies concentrated on general populations, outbreak cohorts, and specific risk categories (11, 19, 27, 29, 42, 53, 54); however, our study examines febrile clinic patients, which is novel and beneficial for public health intervention strategies. Using both A35R and A29L strengthens the specificity of serological detection, as these antigens are known to elicit robust antibody responses following MPXV exposure. A35R is an extracellular enveloped protein, while A29L intracellular mature protein. These MPXV immunogenic proteins are well documented for their robust specificity for MPXV and orthopox viruses. Their combined use in a Luminex multiplex format enhances assay performance and supports applicability for surveillance in endemic or resource-limited settings where seroepidemiological surveillance is highly needed.

Several limitations include the cross-sectional design and the convenience sampling technique, which prevents inference about the timing of infection and generalizability of our findings. The prevalence estimate may not be generalized nationally, and the finding is exploratory and surveillance-based. We used a limited number of positive and negative controls to model antibody signal distributions. Although the repeated measurement of the four positive controls increases assay precision, it does not increase the biological diversity of positive controls and may also limit the statistical power of the seropositivity modeling and classification. Consequently, the estimated distributions may not fully represent the variability in antibody responses across the broader population. Our dataset did not include participants’ exposure history, such as contact with infected individuals, previous mpox infection, animals, vaccination, and travel history; hence, it was impossible to differentiate between exposed and unexposed individuals and to assess the relationship between exposure and seropositive status. We also acknowledge that the potential generalization of our findings is limited because of the selection bias introduced by our convenience sampling approach.

Although these antigens improve specificity, cross-reactivity with other orthopoxviruses cannot be fully excluded. Additionally, the study population consisted of febrile patients attending healthcare facilities and may not represent the general population.

## Conclusion and recommendations

Our study demonstrates measurable MPXV seroprevalence among febrile patients attending healthcare facilities in four Nigerian states. The use of A35R and A29L antigens identified substantial evidence of exposure, supporting integration of serological surveillance into existing mpox monitoring systems. Larger longitudinal studies incorporating exposure histories and molecular diagnostics are needed to clarify transmission pathways and quantify the true burden of MPXV exposure in Nigeria.

Given Nigeria’s recurrent MPXV outbreaks and limited molecular diagnostic resources, integrating serology into existing surveillance systems could enhance outbreak preparedness and response. A multiplex serological assay could be incorporated into sentinel surveillance of febrile illness or other infectious disease surveillance to monitor orthopox exposure alongside other emerging pathogens. Apart from complementing PCR, serosurveys could detect changes in population-level exposure, identify previously undetected exposure, and inform targeted public health interventions and risk communication. The finding underscores the need for enhancing the integration of serological, molecular, and genomic approaches in MPXV surveillance in Nigeria. Serological methods can identify evidence of past exposure and estimate the true burden of infection; hence, they can complement molecular methods that timely confirm active infection and genomic sequencing that provides valuable information on viral evolution, transmission dynamics, and new lineages. We recommend integrating MPXV serological surveillance using A35R and A29L into existing mpox monitoring frameworks in Nigeria. Public health authorities should consider incorporating Luminex immunoassays into routine surveillance among febrile patients to enhance detection and tracking of exposure. Larger longitudinal and region-specific studies are needed to better define temporal and geographic trends in seroprevalence and to evaluate antigen performance across diverse populations.

## Data Availability

The original contributions presented in the study are included in the article/Supplementary material, further inquiries can be directed to the corresponding author.
